# Supplementary material for: A comparison of the Netherlands, Norway and UK familial hypercholesterolemia screening programmes with implications for target setting and the UK’s NHS long term plan
Source: PLOS Glob Public Health. 2023 Apr 25;3(4):e0001795. doi: 10.1371/journal.pgph.0001795 (PMC10128934; doi:10.1371/journal.pgph.0001795)
Supplement: Attachment — Submitted filename: PLOS_RebuttalLetter.pdf [file pgph.0001795.s001.pdf]

Personalised Medicine Centre,
School of Medicine, Ulster University,
C-TRIC Building, Altnagelvin Area
Hospital, Derry, Northern Ireland,
BT47 6SB, UK

16th March, 2023

Dear Editor

We would like to thank the reviewers for the considered comments and feedback. In particular, we were delighted to read that the reviewers consider the work to be timely and relevant and found our conclusions to be robustly data driven.

The editorial office requested the following amendments which we have addressed

1. Please amend your detailed Financial Disclosure statement. This is published with the article. It must therefore be completed in full sentences and contain the exact wording you wish to be published.
a. State the initials, alongside each funding source, of each author to receive each grant.
b. State what role the funders took in the study. If the funders had no role in your study, please state: "The funders had no role in study design, data collection and analysis, decision to publish, or preparation of the manuscript."
c. If any authors received a salary from any of your funders, please state which authors and which funders.

The financial disclosure now reads,
"This work was supported by funding from the Department for the Economy, Northern Ireland, to all authors. It was also supported by funding from the Public Health Agency (PHA) of the Health and Social Care (HSC) R&D Division and the Western Health & Social Care Trust (to TSR with award number COM/5618/20). The funders had no role in study design, data collection and analysis, decision to publish, or preparation of the manuscript."

Reviewer 1 asked a number of questions of the manuscript that we would like to take the opportunity to address. Reviewer 2 requested no changes.

Reviewer #1: The article dealt with an issue of timely relevance and put forward sound data-backed conclusions.

However I have a few questions on the article which I am stating below.

1.Authors have chosen Norway and Netherlands as countries for comparison. I would welcome some references which establish that these are the countries with well established screening programs, which is the reason authors have given for selecting these particular countries.

On page 5, in the final paragraph of the introduction, we have added two references to papers published on behalf of from the European Atherosclerosis Society that

highlight the significance of the Norway and Netherlands screening programmes ([16, 17]).

2.In Page 11 line 223 its said that number of FH positive relatives identified per index case is 2.12., 2.52 and 3.24 for Norway , Netherlands and Northern Ireland respectively. The same values are also given in Table 2. However, in line 225 the values mentioned for FH positive relatives identified per index case is 2.74(Norway), 3.12(Netherlands) and 3.24(Northern Ireland). Why is there a (slight) discrepancy and what is the source of the latter set of values?

Our apologies, the latter values were from an earlier, less accurate calculation and have now been corrected.

3.In Table 2, Relatives identified with FH per index case is not equal to the ratio of Relatives identified with FH after DNA testing and Index cases identified; for United Kingdom. The value given in the table is 0.99 but the value calculated from the latter two columns comes to 1.12.

Thank you for catching this error. Based on a previous calculation the figures on this row of the table were out of date. The new value is 1.14 and we have updated this in the table and throughout the manuscript. We have also double checked all tables throughout the manuscript to remove any other errors.

4.In line 186, it is mentioned that The LTP sets the target of identifying at least 25% of the FH population of England by 2024 . However, we can see in Figure 4 that this will be unsuccessful if detection continues at the upper estimate of 0.27% of cases per year. However, Figure 4 just shows proportion of FH cases identified without making explicit the rate of detection or time period.

We acknowledge that this is not made clear in the manuscript. We think that reviewer 1 is referring to Figure 3 and Figure 4 and this is not made clear for Figure 4. We have amended the caption to indicate that this bar chart describes the latest reported figure for each country and we refer to the primary data in table 1.

Reviewer #2: The paper is very readable and is of interest in formulating a national screening program. Screening for Familial hypercholesterolemia is now a Class I recommendation and in this context comparison of well established screening programs is commendable

In addition to the above corrections, we have amended a several minor typographic and referencing errors.

Thank you for considering this manuscript for publication in PLoS Global Public Health.

Kind regards

Mr. Christopher M. Page
On behalf of all authors.
